# Haplotype reconstruction using perfect phylogeny and sequence data

**DOI:** 10.1186/1471-2105-13-S6-S3

**Published:** 2012-04-19

**Authors:** Anatoly Efros, Eran Halperin

**Affiliations:** 1 The Blavatnik School of Computer Science, Tel-Aviv University, Tel-Aviv, Israel; 2 International Computer Science Institute, Berkeley, California, USA; 3 Department of molecular microbiology and Biotechnology, Tel-Aviv University, Tel-Aviv, Israel

## Abstract

Haplotype phasing is a well studied problem in the context of genotype data. With the recent developments in high-throughput sequencing, new algorithms are needed for haplotype phasing, when the number of samples sequenced is low and when the sequencing coverage is blow. High-throughput sequencing technologies enables new possibilities for the inference of haplotypes. Since each read is originated from a single chromosome, all the variant sites it covers must derive from the same haplotype. Moreover, the sequencing process yields much higher SNP density than previous methods, resulting in a higher correlation between neighboring SNPs. We offer a new approach for haplotype phasing, which leverages on these two properties. Our suggested algorithm, called *Perfect Phlogeny Haplotypes from Sequencing *(*PPHS*) uses a perfect phylogeny model and it models the sequencing errors explicitly. We evaluated our method on real and simulated data, and we demonstrate that the algorithm outperforms previous methods when the sequencing error rate is high or when coverage is low.

## Introduction

The etiology of complex diseases is composed of both environmental and genetic factors. In the last few decades, there has been a tremendous effort to discover the genetic component of the etiology of a large number of common traits, that is, characterizing the heritability of these traits. In recent years, much of this effort has been focused on genome-wide association studies (*GWAS*), in which the DNA of a population of cases (individuals carrying the studied condition), and a population of controls (general population) is being measured and compared. These studies have been focusing on measurements of single nucleotide polymorphisms (*SNPs*), which are positions in the genome in which at some point in history there has been a mutation that was fixed in the population.

In recent years, genotyping technology, or the extraction of the SNP information from the genome, has been advancing rapidly. Only a few years ago, genome-wide association studies were simply infeasible. On the other hand, even the most modern genotyping technologies only provide a partial picture of the genome, since the number of positions measured with these technologies is typically less than a million, while there are more than three billion positions in the genome. Additionally, there are many different types of genetic variations that are not captured well by genotyping technologies, particularly rare SNPs, and short deletions and insertions. For this reason the next generation of genetic studies of diseases will surely include the new high-throughput sequencing technologies, or next generation sequencing platforms (*NGS*). These technologies provide in one experiment hundreds of millions of short sequence reads of the sampled DNA.

The technical analysis of disease association studies encountered a few computational challenges, some of which will remain when considering NGS based studies. One of the major obstacles in these studies has been the inference of haplotypes from the genotype data (*phasing*). As opposed to genotypes, a haplotype is the sequence of alleles across a chromosome. Genotype technologies provide the information about the number of minor alleles occurring at each position, but not the relation between the positions. Mathematically, we can think of a haplotype as a binary string, and the genotype is simply the sum of the two haplotypes of the corresponding chromosomes. In the last decade, many phasing algorithms have been suggested [1-7]; these algorithms take as input a set of genotypes, and leverage the correlations between neighboring SNPs, or the linkage disequilibrium (*LD*), to infer the haplotypes.

The phasing problem is of different nature when applied to sequencing studies. First, unlike genotyping technologies, sequence data allows us to consider both SNPs and short structural variations (e.g., short deletions). Second, in sequencing technologies the measured SNPs are closer to each other than in genotyping, resulting in a much higher LD, or correlations between neighboring SNPs. Third, the short reads obtained from the sequencing platform are always read from one chromosome, and some of these reads may contain more than a single SNP, suggesting that partial haplotypes are provided. Finally, the noise obtained by NGS technologies is inherently different than genotyping technologies; the sequence reads contain substantially more errors than genotypes, especially towards the end of the reads, and the final errors made by the algorithms are highly dependent on specific parameters such as the *coverage*, which is the expected number of reads overlapping with a specific position in the genome.

Unlike the case of genotyping, sequencing allows the possibility of phasing a single individual (genotype data requires a population). Very recently, a few methods were suggested for the problem [8,9]; these methods leverage the fact that each sequenced read originates from a single chromosome, and that all variants covered by the read must originate from the same haplotype. They use these sets of partial haplotypes to reconstruct a full haplotype.

## Methods

The basic assumption of our algorithm is that within a short region, the history of the genetic variants (SNPs or deletions) follows the perfect phylogeny model, i.e., there are no recurrent mutations or recombinations. We denote by *T = *(*V*, *E*) the underlying directed perfect phylogeny tree, where each *v *∈ *V *corresponds to a haplotype of length *m *(a string over {0,1}*^m^*) and every edge corresponds to a mutation. For every *v *∈ *V*, let *p_v _*be the haplotype frequency in the population.

### Modeling the sequencing procedure

The haplotypes themselves are not given by the sequencing procedure, but instead, the sequencing procedure provides a large set of short reads, arguably sampled from random positions in the genome and from a random copy of the chromosome (out of the two copies). The sequencing itself is a noisy procedure, which depends on two parameters, the coverage *k*, the read length *l*, and the sequencing error rate *ε*. We will assume each read is a copy of *l *bases extracted from the genome starting at a random position. The copy is not an exact copy and we will assume that there is a probability *ε *for the base to be read incorrectly. Under simplifying assumptions, we can assume that the error rate does not depend on the genomics position.

### Problem statement

The input for our algorithm is the sequence data, i.e., the set of reads obtained from the sequencer, where each read is assumed to be generated by randomly picking a position in the genome, randomly picking one of the copies of the chromosome in that position, and adding noise using the parameter *ε *in each position of the read independently. We will denote the set of reads of individual *i *as *R_i_*. Additionally, we will denote the two haplotypes of individual *i *as $H_{i}=\left(h_{i}^{1},h_{i}^{2}\right),$ where $h_{ij}^{1},h_{ij}^{2}$ are the alleles of the haplotypes in SNP *j*, and $h_{i,S}^{1},h_{i,S}^{2},$ are the haplotypes restricted to the set of SNPs *S*. If *H_i _*is known for each *i*, we can write the likelihood of the data as

$$\text{Pr}\left(R_{1},...,R_{n}|H_{1},...,H_{n}\right)=\overset{n}{\underset{i=1}{\prod }}\underset{r\in R_{i}}{\prod }\text{Pr}\left(r|H_{i}\right),$$

where *Pr *(*r|H_i_*) is the probability of observing read *r *given the two haplotypes of individual *i*. Particularly, if *r *spans the positions of a set of SNPs *S*, and if *d*(*x*, *y*) is the Hamming distance between two sequences *x *and *y *(restricted to the set of SNPs), then

(1) $$\mathrm{Pr}\left(r|H_{i}\right)=\frac{1}{2}\varepsilon^{d\left(h_{i,S}^{1},r\right)}{\left(1-\varepsilon \right)}^{\left|S\right|-d\left(h_{i,}^{1}{}_{S},r\right)}+\frac{1}{2}\varepsilon^{d\left(h_{i,S}^{2},r\right)}{\left(1-\varepsilon \right)}^{\left|S\right|-d\left(h_{i,}^{1}{}_{S},r\right)}$$

Our algorithm aims at finding a perfect phylogeny tree on the set of SNPs in a given window, and a corresponding haplotype assignment for each individual. The haplotypes assigned to each individual need to be taken from the tree, and the objective is to optimize the likelihood of the reads. We will explain later how the perfect phylogeny assumption can be relaxed.

### Tree reconstruction within a window

Within a window, the tree reconstruction algorithm works in the following way. We first search for a SNP j* that is adjacent to the root of the tree. We then use a partitioning procedure to decide for every other SNP *j*, whether it is a descendent of j* in the tree. This partitioning procedure splits the set of SNPs into two, and we recursively compute the two subtrees.

Throughout, we assume that the alleles of each SNP are represented by the {0, 1} notation, where 0 is the more common allele. As shown in [10], the set of haplotypes corresponds to a perfect phylogeny, if and only if it corresponds to a perfect phylogeny with the all zeros vector as the root. We thus assume that the root of the tree is the haplotype with all 0 values. Under these assumptions, it is clear that for two SNPs *j *and *j'*, SNP *j *cannot be a descendant of SNP *j' *if their corresponding allele frequencies satisfy *f_j _> f_j'_*. Therefore, we first estimate the minor allele frequency of each SNP and we choose the SNP j* as the SNP with the largest estimated allele frequency, breaking ties arbitrarily.

For a SNP *j *and an individual *i*, we denote by *R_ij _*⊆ *R_i _*the set of reads that overlap with SNP *j*. We write the likelihood of the minor allele frequency as follows:

$$ℒ\left(f_{j};R_{1j},...,R_{nj}\right)=\overset{n}{\underset{i=1}{\prod }}\left(\overset{2}{\underset{g=0}{\sum }}\left(f_{j}^{g}{\left(1-f_{j}\right)}^{2-g}2^{\left(2-g\right)g}\underset{r\in R_{ij}}{\prod }\text{Pr}\left(r|G_{ij}=g\right)\right)\right),$$

where $G_{ij}=h_{ij}^{1}+h_{ij}^{2}$ is the genotype of individual *i *and position *j*. Note that Pr(*r | G_ij_*) is given in Equation (1), restricted to the case *S *= {*j*}. Now, $A_{i,j,g}=\prod_{r\in r_{ij}}\text{Pr}\left(r|G_{ij}\right)$ can be computed in linear time, in a preprocessing procedure, regardless of *f_g_*. Therefore, the log likelihood can be rewritten in the following way:

$$\text{log}\left(ℒ\left(f_{j};\;R\right)\right)\;=\;\overset{n}{\underset{i\;=\;1}{\sum }}\text{log}\;\left(\overset{2}{\underset{g=0}{\sum }}A_{i,j,g}f_{j}^{g}{\left(1\;-\;f_{j}\right)}^{2-g}2^{\left(2-g\right)g}\right),$$

We use an expectation-maximization [11] (*EM*) algorithm to estimate *f_j_*.

### The partitioning procedure

Now that we have chosen j*, the algorithm proceeds by partitioning the other SNPs into two sets, *T*_1 _and *T*_2_, where *T*_1 _corresponds to the subtree of the root excluding the edge j* and its descendants, and *T*_2 _corresponds to the subtree located below j*. In order to nd this partition, for each SNP *j*, we calculate the likelihoods of *j *being in *T*_1 _verses the likelihood of *j *being in *T*_2_, and we assign *j *to the tree for which the likelihood is higher. We later refer to this partitioning method as PPHS-2.

**Figure 1:**
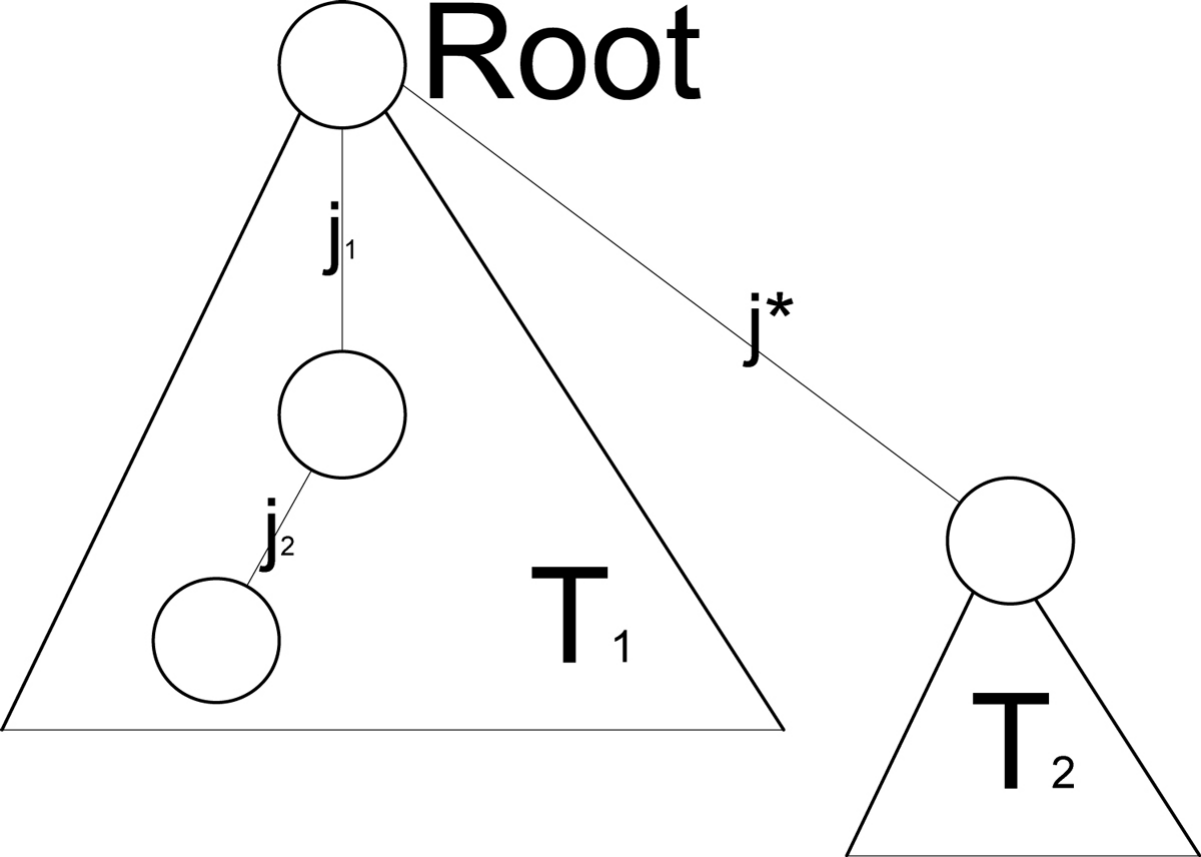
**Graph separation example**. One out of 3 different options of Tree T* where *j*_2 _is the child of *j*_1_.

This approach is highly efficient (the running time of each partition iteration is linear), however, the algorithm is highly sensitive to mistakes occurring early on in the process of the tree reconstruction, and it does not take into account the overall multivariable relations between j* and all other SNPs. For this reason, we also consider an alternative partitioning approach, (PPHS-3), which is based on the pairwise relations of pairs of SNPs with j*. Formally, for each *j*_1_, *j*_2_, ∈ {1,..., m}\{ j*}, we consider the four possible configurations, that is (1) *j*_1_, *j*_2 _∈ *T*_1_, (2) *j*_1_, *j*_2 _∈ *T*_2_, (3) *j*_1_, ∈ *T*_1_, *j*_2 _∈ *T*_2_, and (4) *j*_1_, ∈ *T*_2_, *j*_2 _∈ *T*_1_. Each of these configurations corresponds to a set of possible subtrees induced by j*, *j*_1_, *j*_2_. Particularly, in cases (1) and (2) there are three possible subtrees, while cases (3) and (4) correspond to a unique subtree (see Figure 1).

For each of these subtrees, T* , we guess the most likely haplotype frequency distribution over the tree using EM. As before, *p_v _*corresponds to the frequency of the haplotype represented by vertex *v*, and *p^t^*(*v*) corresponds to the guess of the EM algorithm at step *t*.

In practice, we start the EM algorithm from a distribution $p_{v}^{0}$ defined as follows. Let *s*_0 _be the SNP corresponding to the parent edge of *v*, and let *S *be the set of SNPs corresponding to the children edges of *v*. We set $p_{v}^{0}=p_{so}-\sum_{s\in S}ps,$ where *p_s _*is the estimation of the allele frequency of SNP *s *using the EM as described above. It is easy to verify that if the allele frequency are accurately estimated then the haplotype frequencies are correctly estimated as well (restricted to the tree T*).

The EM algorithm provides us with a set of haplotype frequencies $p_{h}^{T^{*}}$ corresponding to the tree T*. The likelihood of the subtree can now be written as

$$\text{Pr}\left(R|T^{*}\right)=\overset{n}{\underset{i=1}{\prod }}\underset{h_{1}h_{2}\in T^{*}}{\sum }\text{Pr}\left(R_{i,\left\{j_{1},j_{2},j^{*}\right\}}|\left(h_{1},h_{2}\right)\right)p_{h_{1}}^{T^{*}}p_{h_{2}}^{T^{*}}$$

We now define a set of four complete graphs *G_ab _*= (*V, E_ab_*) (*a, b *∈ {1,2}) where *V *is the set of SNPs. In graph *G_ab_*, an edge *e*_j1,*j*2 _∈ *E_ab _*has an associated weight

$$w_{ej1,j2}^{ab}=\frac{1}{|\left\{T^{*}|j1\in T_{a},j2\in T_{b}\right\}|}\underset{T^{*}\in \left\{T^{*}j1\in T_{a},j2\in T_{b}\right\}}{\sum }\text{Pr}\left(R|T^{*}\right)$$

Let (*S*_1_, *S*_2_) be a partition of the graphs *G_ab_*. Let

$$w_{ab}\left(S_{1},S_{2}\right)=\underset{j1\in S_{a},j2\in S_{b}}{\sum }w_{\left(j1,j2\right)}^{ab}$$

The algorithm proceeds by searching for the partition that maximizes ∑_*a, b *_*w_ab _*(*S*_1_,*S*_2_). In order to find the best partition, for low values of *m *we enumerate over all possible partitions, and for larger values of *m *we randomly pick subsets of the vertices, compute the partition for these subsets, and use a majority vote to decide on the overall partition.

### Generation of haplotypes for each window

For every window, the recursive process described above returns the perfect phylogeny tree *T *which best fits the data. Now the algorithm needs to select for every individual *i *two haplotypes *h*_1_; *h*_2_. Since the data may not perfectly fit the model, we add additional haplotypes to the pool of possible haplotypes. Using the tree *T *we create a list *L *of haplotypes, where each haplotype *h *corresponds to a specific node *v *∈ *T*. From each haplotype in *L *we create m new haplotypes, where *m *is the number of SNPs in the window by adding all possible one-mutations to the haplotype. We call these haplotypes the haplotypes derived from *L*.

For each individual *i *we select two haplotypes *h*_1_, *h*_2 _which maximizes the a posteriori probability:

$$\text{Pr}\left(h_{1},h_{2}|R_{i}\right)\alpha \text{Pr}\left(R_{i}|h_{1},h_{2}\right)\text{Pr}\left(h_{1}h_{2}\right)=\text{Pr}\left(h_{1}\right)\text{Pr}\left(h_{2}\right)\underset{r\in R_{i}}{\prod }\text{Pr}\left(r|h_{1},h_{2}\right)$$

The priors Pr(*h*) are calculated based on the haplotypes assigned so far to other individuals, and based on the fact that the haplotypes in *L *are more likely than the haplotypes derived from *L*. Particularly, when we consider individual *i *+ 1, let *H_i _*be the set of haplotypes assigned to the first *i *individuals, and for each haplotype *h*, let *n_h _*be the total number of occurrences of *h *in *H_i_*. Then, we set the vector of prior to be $\vec{p}=\left({p_{h}}_{{}_{1}},...,p_{h_{k}}\right)$ which maximizes the maximum a posteriori (MAP) under a Dirichelet prior with α_1 _weight in the *L *haplotypes and *α*_2 _weight in the haplotypes derived from *L*:

$$\text{Pr}\left(\vec{p}|H_{i}\right)\alpha \text{Pr}\left(H_{i}|\vec{p}\right)\text{Pr}\left(\vec{p}\right)\alpha \underset{h}{\prod }p_{h}^{nh+\alpha h-1},$$

which is maximized for *p_h _*∝ *n_h _*+ *α_h _*- 1.

### Stitching of windows

The framework discussed so far assumes that the haplotypes are inferred within a window of *m *SNPs. The length of the window is mainly determined by the assumption of perfect phylogeny, and therefore we cannot apply our method to long windows. When working on large number of SNPs we use BEAGLE results as a guide that connects the different windows.

Let $h_{i_{1}}^{\prime },h_{i_{2}}^{\prime }$ be the haplotypes returned by BEAGLE for individual *i*. Consider a window *w *or length *m *spanned by SNPs *j *∈{*w*...*w *+ *m *- 1}. Let $h_{i_{1}}^{w},h_{i_{2}}^{w}$ be the haplotype returned by the above algorithm for window *w *and let $h_{i_{1}}^{w^{\prime }},h_{i_{2}}^{w^{\prime }}$ the haplotypes returned by BEAGLE restricted to the same window *w*.

We calculate the Hamming distances *d*_1_, *d*_2_:

$$\begin{array}{ccc} d_{1} & =d\left(h_{i1}^{w},h_{i1}^{w^{\prime }}\right)+d\left(h_{i2}^{w},h_{i2}^{w^{\prime }}\right) & \\ d_{2} & =d\left(h_{i2}^{w},h_{i1}^{w^{\prime }}\right)+d\left(h_{i1}^{w},h_{i2}^{w^{\prime }}\right) \end{array}$$

In case *d*_1 _<*d*_2_, *d*_1 _≤ d* we use haplotypes $h_{i_{1}},h_{i_{2}}$ as the haplotypes for window *w*, if *d*_2 _<*d*_1_, *d*_2 _≤ d* we use haplotypes $h_{i_{1}},h_{i_{2}}$ as the haplotypes for window *w*. In all other cases we use the haplotypes $h_{i_{1}}^{\prime },h_{i_{2}}^{\prime }$ which were returned by BEAGLE as the halotypes for window *w*. Put differently, we use the BEAGLE haplotype results in order to determine the ordering of our solution if our solution is relatively close to BEAGLE's solution. If our solution is far from BEAGLE's solution we assume that the perfect phylogeny model does not hold and therefore we simply use BEAGLE's solution in this case. We chose the threshold d* to be *m/2 *based on empirical evaluation, with this paramters the algorithm choose PPHS answer over BEAGLE's in 60-70% of the windows while working on small populations.

## Results

In order to evaluate the performance of our methods, we first used simulated data to generate a large number of trees, and we measured the accuracy of the tree reconstruction directly. We then considered a set of haplotypes, either randomly generated from the simulated tree, or taken from real data, and we evaluated the phasing accuracy on these data. For the latter, we used an extension of the switch distance error metric. Generally, there are two types of errors; the first type is regular switch errors (for example two haplotypes 11 and 00 are phased as 10 and 01), and the second type is mismatch errors (two haplotypes 11,00 are phased as 11 and 01). We used the sum of all switch and mismatch errors (*S *+ *M *when *S *is the number of switch errors in the data and *M *is the number of mismatch errors). We refer to this as the SM error metric. While running Beagle we used the default software parameters which are 10 iterations. The likelihoods for each SNP *k *were calculated using the following equations:

$$\text{Pr}\left(R_{1},...,R_{n}|H_{1},...,H_{n}\right)=\overset{n}{\underset{i=1}{\prod }}\underset{r\in R_{i}}{\prod }\text{Pr}\left(r|H_{i}\right),$$

Where all reads and haplotypes were restricted to SNP *k *for all options of *H*_i _∈ {(0, 0), (1, 0), (1, 1)}.

### Evaluation under Simulated data

We used the MSMS software [12] to simulate data from a perfect phylogeny. MSMS [12] uses the Coalescent process to create haplotypes and when the mutation rate is set low enough, the resulting haplotypes fit the perfect phylogeny model.

### Accuracy of tree reconstruction

**Figure 2:**
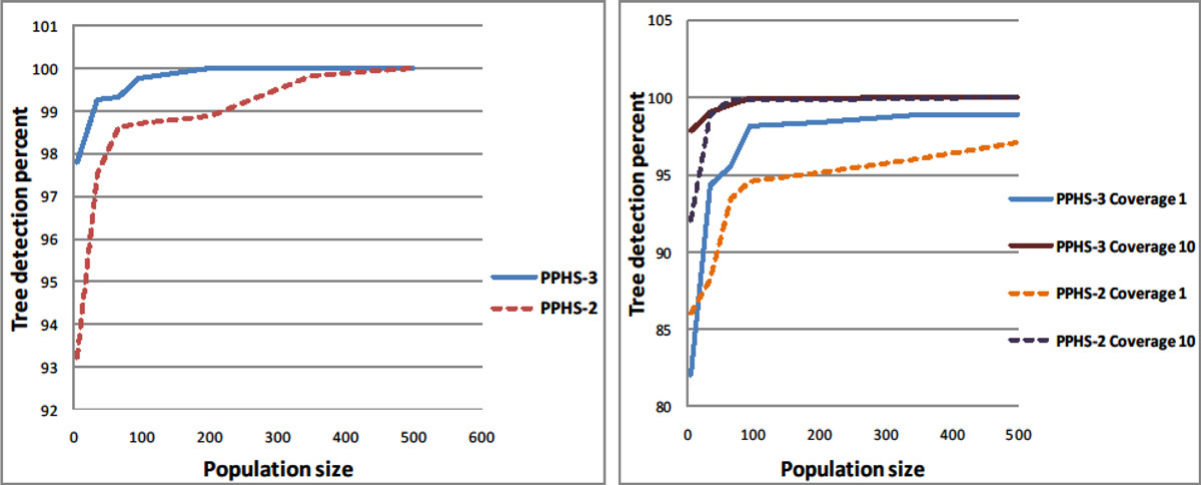
**Tree reconstruction precision**. The tree reconstruction percentage as a function of the population size. The tests were done with 30 SNPs and a minimal haplotype frequency of 3%. On the right (Figure 2.2), the sequencing error rate was 2%, with varying coverage values, and on the left (Figure 2.1), the coverage was 10, and the error rate was set to 4%.

Using MSMS, we generated a set of 100 random haplotype groups over 30 SNPs, and for each group we randomly generated a set of *N *genotypes, where *N *varies from 5 to 500. We then simulated the sequencing procedure on these genotypes (see Methods). For a given set of genotypes, we let PPHS reconstruct the tree, and the set of haplotypes obtained can be compared to the haplotypes of the original tree. In the true tree there are 30 SNPs and therefore there are exactly 31 haplotypes, denoted by *H_True_*. We denote the haplotype frequencies of *H_True _*as *p*_1_,..., *p*_31_. The output of the algorithm results in another tree with a possibly different set of haplotypes, *H_PPHS_*. Let *S *= *H_True _*∩ *H_PPHS_*. Then, we measure the accuracy of the reconstruction as ∑_*i*∈*S *_*p_i_*. We observed (Figure 2) that the reconstruction of the tree by our algorithm is highly accurate, and it converges to the correct tree when the number of samples increases, even in the presence of high sequencing errors.

### Evaluation of phasing accuracy

**Figure 3:**
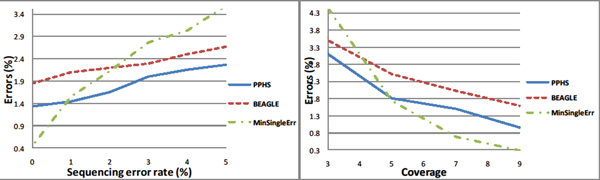
**Phasing accuracy on simulated data**. The window based SM error metric as a function of the sequencing error rate (Figure 3.1) and as a function of the coverage (Figure 3.2). The tests were done with haplotype windows of length was 5 over a population of 5 individuals. The coverage was set to 5 on the left, and the sequencing error rate was set to 3% on the right, each point is the average of 10000 windows.

We compared the results of the PPHS algorithm to BEAGLE [7] (see Figure 3). In addition, in order to compare PPHS to the sequencing based algorithms for phasing, we implemented an algorithm MinSingleErr. In MinSingleErr we compute the likelihood for each genotype at each site, and decide whether the site is heterozygous. If the site is heterozygous, it correctly guesses the phasing in this site. Thus, any haplotype assembly method that uses one sequence at a time (in contrast to a population of individuals) cannot do better than MinSingleErr, and the error rates and no-call rate of MinSingleErr is a lower bound on the no-call rate and error rate of methods such as HapCut and HapSAT [8,9]. We observed that both algorithms show similar behavior for low error rates, however PPHS is more accurate than BEAGLE as the error rate increases. As a function of the coverage, as expected, the performance of all algorithms drastically decreases when the coverage is low. As we can see PPHS maintains a constant improvement over BEAGLE of 20%-40%. If we use the stitch function and compare the SM errors over the entire haplotype our improvement over BEAGLE drops to 10%.

### Evaluation under real data

In the above section we evaluated the performance of the algorithms under the Coalescent model without recombinations. In practice, there are cases in which this model does not capture the empirical behavior of the data and it is therefore important to test the algorithm using real datasets. To do so, we used the EUR population data from the 1000 genome project [13] (taken from the BEAGLE website, 2010-08), in which the full sequences of 283 unrelated individuals of European ancestry are given. For each test we used haplotypes from human chromosome 22 which contains overall 162027 SNPs. We note that the genotype error rate in this data is quite high since the data used for the SNP calling is of very low coverage (phase 1 of the 1000 genomes project). Therefore, it may be the case that in some cases the perfect phylogeny assumption does not hold while in fact the inconsistency is caused by the sequencing errors.

The genotype data provided by the 1000 Genomes project provides us with a realistic setting of the haplotype distribution in the population. Our experiments involved taking these haplotypes and simulating the sequencing process, as described in the Methods. We first measured the accuracy of the algorithms as a function of the coverage and the sequencing errors with a set of five individuals. We also compared our algorithm to haplotype assembly algorithms (HapCut [9]) which work on a single individual. We observed that only in the cases where the coverage is high and the error rate is low do the haplotype assembly algorithms have a chance of preforming better than PPHS and BEAGLE (assuming long reads).

**Figure 4:**
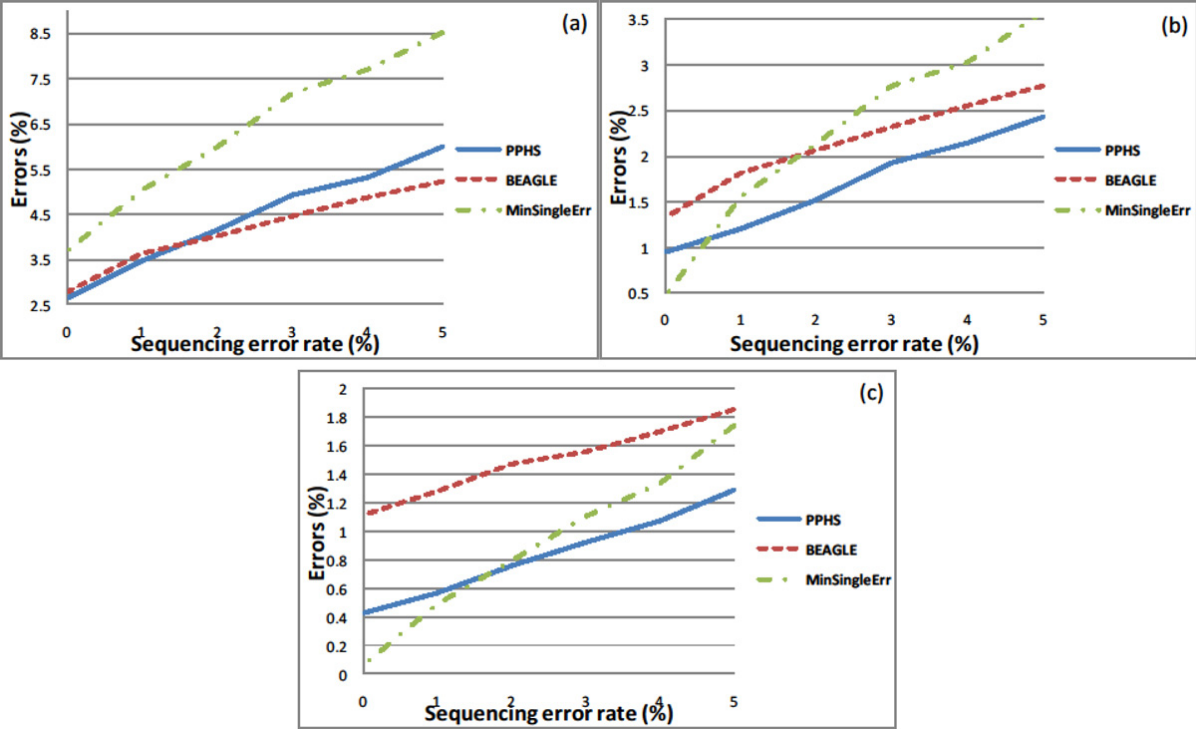
**Phasing accuracy on read data with varying coverage values**. The window based SM error metric as a function of the sequence error rate for coverage values of 2 (Figure 4.1),4 (Figure 4.2),6 (Figure 4.3). Read length was set to 400. The test was done with a population of 5 individuals. The windows size which was used by PPHS is 5 SNPs.

We observed that with coverage of 4,6 PPHS is constantly better than BEAGLE by 40%-100% when using the window based error function (see Figure 4). The advantage over BEAGLE drops to 10% when using the stitch function for PPHS and comparing the error rate over the entire haplotype.

We also compared our algorithm to HAPCUT, as can be seen in table 1, but since HAPCUT requires long reads and high coverage to obtain quality results, its results were far worse than PPHS in our tests.

**Table 1:** The performance of HapCut versus PPHS.

Algorithm	Test 1	Test 2	Test 3	Test 4
HapCut - No Calls	20.85	20.63	18.67	18.03

HapCut - Errors	15.92	8.71	24.80	15.56

Beagle	2.76	1.81	2.20	1.09

PPHS	2.43	1.20	1.71	0.24

All tests were done with 5 individuals, tests 1-3 had an expected coverage of 5 while test 4 had an expected coverage of 20. Tests 1,3 had a sequencing error rate of 5% and tests 2,4 had a sequencing error rate of 1%. The read length of tests 1,2 was 400 while for test 3,4 it was 2000 bases. The cells are the percent of Switch-Mismatch errors in the data.

## Conclusions

This work presents a new algorithm for phasing. This algorithm works by reconstructing the prefect phylogeny tree in every short region. Unlike previous methods (e.g. HAP [5]) we use raw read data to build the tree. Since raw read data includes sequencing errors, methods to overcome them were developed. The fact that a single read can contain more than one SNP is also used by the algorithm. Some deviation from perfect phylogeny is allowed by permitting a single recurrent mutation event in each haplotype.

The results demonstrate that the proposed algorithm works well and is immune to sequencing errors and small population sizes, making it robust. In addition to the solution for the phasing problem, the algorithm provides a new method to reconstruct perfect phylogeny under the condition of an error model. This approach, however, has a few limitations, which may be of interest for further research. The method is designed to work in windows. In order to stitch the windows the BEAGLE results are used as a skeleton, and it is likely that more tailored methods (e.g. a variant of [Halperin, Sharan, Eskin] [14]) may perform better.

An additional limitation of the algorithm is its performance while handling large populations. As the results show, the performance of BEAGLE improves rapidly with the size of the population, whereas the performance of PPHS improves at a slower rate. However, given simulated data (based on the Coalescent model), the algorithm's performance does improve considerably as the population size is increased. This might suggest that when using the 1000 genomes data, the perfect phylogeny model breaks as the number of samples increases due to subtle population structure, or simply large number of errors within that data. If the former is true, there may be an optimization procedure that selects the length of the windows as a function of the linkage disequilibrium structure in the region. Such optimization of the parameters may result in better phasing algorithms, and particularly in a better reconstruction of the trees.

## Competing interests

The authors declare that they have no competing interests.

## Authors' contributions

AE and EH developed the method. AE and EH designed the experiments. AE implemented the method and performed experiments. AE and EH analyzed results and wrote the manuscript. All authors read and approved the final manuscript.
